# A High-Throughput Screen against Pantothenate Synthetase (PanC) Identifies 3-Biphenyl-4-Cyanopyrrole-2-Carboxylic Acids as a New Class of Inhibitor with Activity against *Mycobacterium tuberculosis*

**DOI:** 10.1371/journal.pone.0072786

**Published:** 2013-11-07

**Authors:** Anuradha Kumar, Allen Casey, Joshua Odingo, Edward A. Kesicki, Garth Abrahams, Michal Vieth, Thierry Masquelin, Valerie Mizrahi, Philip A. Hipskind, David R. Sherman, Tanya Parish

**Affiliations:** 1 Seattle Biomedical Research Institute, Seattle, Washington, United States of America; 2 TB Discovery Research, Infectious Disease Research Institute, Seattle, Washington, United States of America; 3 Institute of Infectious Disease and Molecular Medicine, University of Cape Town, Cape Town, South Africa; 4 Lilly Research Laboratories, Eli Lilly & Company, Indianapolis, Indianapolis, United States of America; The Ohio State University, United States of America

## Abstract

The enzyme pantothenate synthetase, PanC, is an attractive drug target in *Mycobacterium tuberculosis*. It is essential for the *in vitro* growth of *M. tuberculosis* and for survival of the bacteria in the mouse model of infection. PanC is absent from mammals. We developed an enzyme-based assay to identify inhibitors of PanC, optimized it for high-throughput screening, and tested a large and diverse library of compounds for activity. Two compounds belonging to the same chemical class of 3-biphenyl-4- cyanopyrrole-2-carboxylic acids had activity against the purified recombinant protein, and also inhibited growth of live *M. tuberculosis* in manner consistent with PanC inhibition. Thus we have identified a new class of PanC inhibitors with whole cell activity that can be further developed.

## Introduction

Nearly one third of the human population is infected with *Mycobacterium tuberculosis*, the causative agent of tuberculosis (TB) [1]. Despite the existence of approved drug regimens against TB, it continues to claim approximately 1.4 million lives every year [1], and the emergence of increasingly drug resistant strains has made the need for improved therapies more urgent.

A hallmark of *M. tuberculosis* is its lipid-rich cell wall, which is an essential element of intracellular survival and pathogenicity, and is also thought to contribute to the difficulty of effectively delivering antimicrobial agents into the cell. The significance of this lipid-rich cell wall is underscored by the large number of genes (∼250) encoding enzymes in fatty acid metabolism present in the *M. tuberculosis* genome [2], making this pathway a promising target for new antibacterial drug discovery. Indeed, several anti-tubercular agents are known to inhibit cell wall biosynthesis.

The *panC* gene encodes the enzyme pantothenate synthetase (PS or PanC), necessary for the production of pantothenate (vitamin B5) in bacteria. Pantothenate is a key precursor for the biosynthesis of coenzyme A (CoA) and acyl carrier protein (ACP), critical components of fatty acid synthesis. The gene encoding PanC is essential for optimal growth *in vitro* [3], and when genetically disrupted in *M. tuberculosis*, the resulting strain is auxotrophic, requiring pantothenate supplementation for growth [4], [5]. Additionally, pathogenicity is severely attenuated in the pantothenate auxotroph [6]. PanC is absent in mammals, who scavenge pantothenate from their diet using pantothenate permease [7], [8], of which there is no homolog in *M. tuberculosis*. This suggests the potential for developing drugs that do not have cross-reactive toxicity to homologs in the host, and makes PanC an attractive drug target in *M. tuberculosis*.

PanC catalyzes the ATP-dependent condensation of pantoate and β-alanine to form pantothenate, simultaneously releasing AMP and pyrophosphate [9]. Previously described assays of PanC activity coupled AMP production to an enzyme cascade that results in the oxidation of NADH to NAD+ in reactions catalyzed by myokinase, pyruvate kinase and lactate dehydrogenase [9]. An enzyme-based screen of *M. tuberculosis* PanC (PanC_MTB_) against a library of 4080 compounds identified a weak *in vitro* inhibitor with no observable whole cell activity [10]. However, because this assay relied on a kinetic measurement, and because absorbance measurements of NADH at 340 nM are often complicated by auto-fluorescence in a compound library, we chose to adapt this assay for high throughput screening. The kinetic assay was modified to generate a fluorescent signal that can be measured as a single time-point (end point assay). We used this assay to conduct a high-throughput screen against a large and diverse compound library, and identified several novel inhibitors of PanC_MTB_, some of which are active against live *M. tuberculosis*.

## Materials and Methods

### Chemicals

Chemicals were obtained from Sigma Aldrich, unless otherwise noted. Pantoate was synthesized as previously described [9]. Myokinase (M3003), pyruvate kinase (P1506), and L-lactic dehydrogenase (L2500) all isolated from rabbit muscle, were purchased from Sigma Aldrich.

### Isolation and purification of recombinant PanC_MTB_ (Rv3602c)

An *Escherichia coli* expression vector (pET28b+) encoding PanC_MTB_ with an amino-terminal 6X-Histidine tag (Dr. Courtney Aldrich, University of Minnesota) was transformed into *E. coli* BL21(ΔE3), grown to mid-log phase and induced with 0.2 mM IPTG at 18°C for 16 hours. A cell lysate was prepared by treatment with lysozyme and sonication; the cleared lysate was applied first to a nickel column to isolate His-tagged proteins, followed by an additional step of purification by size exclusion chromatography (HisTrap and Sepharose 200, Amersham). PanC_MTB_, purified to apparent homogeneity by SDS-PAGE, was concentrated to 2–5 mg/mL in 50 mM HEPES, 50 mM NaCl, 5 mM MgCl_2_ and 5% glycerol, flash frozen and stored at −80°C until use.

### Low-throughput assay for PanC_MTB_ activity- kinetic NADH depletion

The activity of recombinant PanC_MTB_ was measured, as previously described [9]. Briefly, compounds or carrier DMSO alone were incubated with PanC_MTB_, the coupling enzymes, and their reagents for five minutes. The reaction was then initiated by addition of the PanC_MTB_ substrates, pantoate and β-alanine. The reaction was conducted in wells of a black clear-bottomed microplate containing a final volume of 40 µL per well. The final concentrations were 0.4 mM NADH, 10 mM ATP, 1 mM pantoate, 5 mM β-alanine, 1 mM phosphoenol pyruvate (PEP-K), 10 mM MgCl_2_, 2% DMSO, 1 µg/mL of PanC, and 18 U/mL each of myokinase, pyruvate kinase and lactate dehydrogenase in 100 mM HEPES pH 7.8. The plate was immediately transferred to a SpectraMax micro-plate reader and the rate of NADH depletion was monitored by measuring the absorbance at 340 nm every 20 s for a total of 20 min.

### High-throughput assay for PanC_MTB_ activity- single-timepoint fluorescence

The assay was run using a Beckman Coulter Core robotic system. Key components included an ORCA arm, Multimek, Nanoscreen, Victor 2, custom deck chiller and custom plate shuttles. The automated components were controlled and scheduled using SAMI software. Assay results were determined using custom software and managed with Collaborative Drug Discovery's (CDD) Laboratory Information Management System (LIMS).

**Figure 1:**
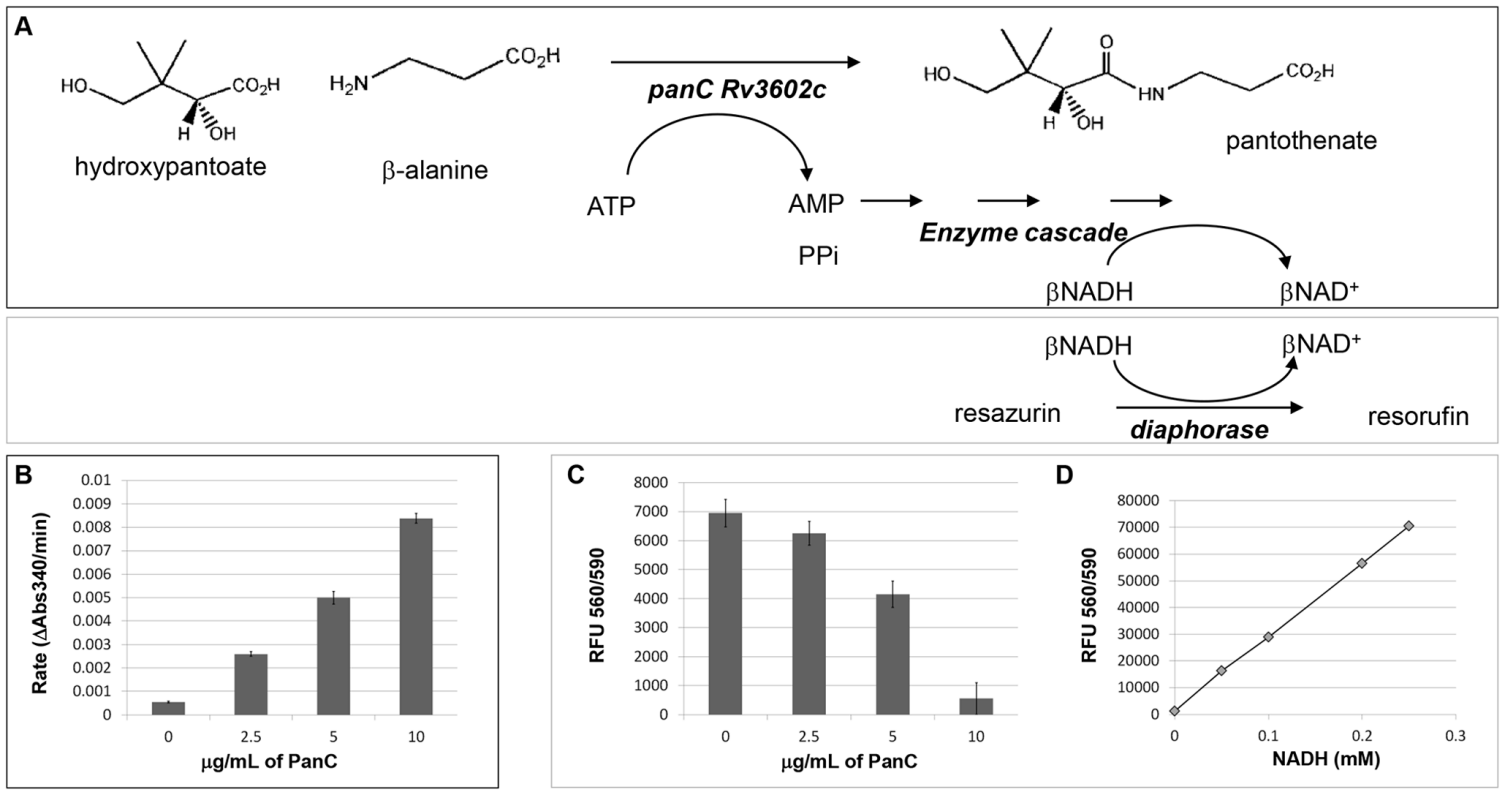
Single-time point fluorescence assay for PanC. **A**) Reaction schematic. Upper panel shows the reaction catalyzed by PanC and the enzyme cascade that is initiated by the reaction product AMP resulting in βNADH oxidation. Lower panel shows the final βNADH dependent fluorescence generating reaction that is coupled to the PanC-initiated enzyme cascade. **B**) Low-throughput assay: kinetic reaction monitoring the rate of βNADH oxidation. **C**) High-throughput assay: fluorescent resorufin signal generated by residual βNADH following the PanC initiated enzyme cascade. **D**) Fluorescence generated with varied βNADH in solution using the same conditions as in (B).

The assay is shown in Figure 1A. The kinetic NADH depletion assay was initiated as described above. After 30 minutes 10 µL of solution containing fluorescent reagents was added to each well. The final 50 µL reaction contained 12.5 U/mL of diaphorase and 5 mM resazurin. After thorough mixing, the plate was transferred to a micro-plate reader and the level of resulting NADH-dependent resorufin determined by measuring fluorescence (excitation and emission filters set at 560 nM and 590 nM, respectively).

### Preparation of compound plates for HTS and for CRC

HTS library sets of individual compounds were plated at 1 mM - diluted first into HEPES and finally into the assay for a final assay concentration (FAC) of 20 µM. For concentration response curves (CRCs), the compounds were plated in single wells at 10 mM and diluted 3-fold across 9 adjacent wells in a row. Serial dilutions were made in 100% DMSO to ensure accurate concentrations of compound across the series. This entire plate was then treated in the same format as the HTS library plates, for FACs ranging from 200 µM- 0.01 µM in 2% DMSO (details provided in Text S1 and shown in Figure S3).

### Analog Retrieval

Analog retrieval from the Lilly collection was performed by chemical fingerprint similarity search (Tanimoto similarity threshold of 0.8). The molecular composite fingerprints used for this study consisted of four components. First, 2048 bits were derived from linear paths similar to Daylight fingerprints, then 160 bits that had their origins in the MACCS keys. A further 160 bits were derived from the second bit vector, but where bits were set only when repeated features were found. In addition, 8 molecular properties were added (atom count, size of largest ring, number of rings, number of ring atoms, number of aromatic atoms, number of fused ring atoms, number of heteroatoms) [11], [12].

### Strains

*M. tuberculosis* H37Rv (ATCC 27294) was used as the parent strain in all experiments (also referred to by the abbreviation RvS). PanC-TetON_M_, a strain in which *panC* (*Rv3602c*) is under the conditional expression of a tetracycline-inducible promoter [5] was used in this study. The strain was maintained and cultured in the presence of 50 µg/mL of hygromycin, 20 µg/mL kanamycin and 50 µg/mL pantothenate supplement (vitamin B5), unless otherwise specified.

### *M. tuberculosis* viability assays

*M. tuberculosis* strains RvS and PanC-TetON_M_ were grown to log phase (OD_600_∼0.3), diluted to a final theoretical OD_600_ of 0.002 and dispensed into a 96-well round-bottom plate (Corning, Acton, MA) in a final volume of 180 µL. To these cells 20 µL of compound diluted in 7H9 and 10% DMSO was added to yield 200 µL (final concentration of 1% DMSO). For each strain, control wells containing no compound were used as a measure of 100% growth, while wells containing a 1∶100 dilution of the starting culture were used as a measure of 99% inhibition. RvS was plated in 7H9 containing 0.2% w/v glycerol, 10% v/v OADC supplement (oleic acid, albumin, D-glucose, catalase; Becton Dickinson) and 0.05% w/v Tween 80 (7H9-GAT) with 0.5 ng/mL anhydrotetracycline (ATc). PanC-TetON_M_ was plated in 7H9-GAT with 12.5 µg/mL of hygromycin, 6.25 µg/mL kanamycin and 0.5 ng/mL ATc, either in the presence or absence of 50 µg/mL pantothenate. Plates were incubated at 37°C for 6 days, cells were resuspended by pipetting and 20 µL of the total cell mixture was used in a Bac-Titer Glo™ (Promega, Madison, WI) assay of cell viability, as per the manufacturer's instructions. Luminescence readings were conducted on a FluoStar Omega plate reader (BMG Lab Tech, Cary, NC). Data from dose-response experiments was represented as the percent inhibition compared with the no-drug controls and analyzed with Graphpad Prism™ (San Diego, CA). The MIC_50_ for each growth condition was calculated by fitting the data to a non-linear least-squares curve.

### Cytotoxicity assay

Cytotoxicity was measured against the African green monkey adult kidney cell line (Vero). Vero cells were plated at 25,000 cells/mL in black 96-well assay plates pre-populated with compound dilutions and controls. Cells were incubated in a humidified 5% CO_2_ environment at 37°C for 48 h; intracellular ATP levels were measured using CellTiter-Glo®Reagent (Promega, Madison, WI) [13]. Luminescence was measured using a Victor 2 plate reader and percentage inhibition of growth calculated. Results were expressed as toxicity concentration (TC_50_) = concentration of compound required to inhibit growth by 50%.

## Results

### Assay description

Recombinant PanC_MTB_ was expressed and purified in *E. coli*. We confirmed the enzymatic activity of the purified enzyme using the kinetic assay [9] (Figure 1B) and then adapted it to be more amenable to high throughput screening. Rather than a kinetic measurement of NADH depletion the level of NADH was determined by the terminal addition of the enzyme diaphorase and its substrate, resazurin, which is converted into the fluorescent dye, resorufin, in an NADH-dependent reaction. In this end-point fluorescence assay the PanC_MTB_ reaction was initiated and then allowed to proceed, depleting the NADH present in solution. After a defined incubation time, while the PanC_MTB_ reaction was still progressing linearly, the diaphorase and resazurin were added, initiating the reduction of the dye with the remaining NADH. We titrated the concentrations of diaphorase and resazurin so that the fluorescent signal developed rapidly and demonstrated linear proportionality to the amount of NADH present in solution (Figure 1D). Therefore, the final fluorescence was inversely proportional to the activity of PanC_MTB_ (Figure 1C).

In a set of representative experiments, the reaction conducted with varying levels of PanC_MTB_ resulted in varied rates of NADH depletion (Figure 1B). The reactions were then coupled to the fluorescence reagents, and levels of the resulting NADH-dependent resorufin measured (Figure 1C). When no PanC_MTB_ was present the fluorescence signal was high. When increasing amounts of PanC_MTB_ were present, the rate of AMP production rose, causing progressive depletion of NADH in the initial reaction, ultimately resulting in a drop in the final fluorescence (Figure 1C). In addition to determining assay conditions under which the fluorescence readout was proportional to levels of active PanC_MTB_, we confirmed that the assay was also proportionately sensitive to varied levels of the substrate, pantoate, by holding the concentration of recombinant PanC_MTB_ constant, but varying the concentrations of pantoate (data not shown). After successfully coupling the kinetic NADH depletion assay to an end-point fluorescent reaction, we optimized it for translation to an HTS format and tested the robustness of this system to confirm that it was HTS-compatible.

### Development and validation for HTS compatibility

**Figure 2:**
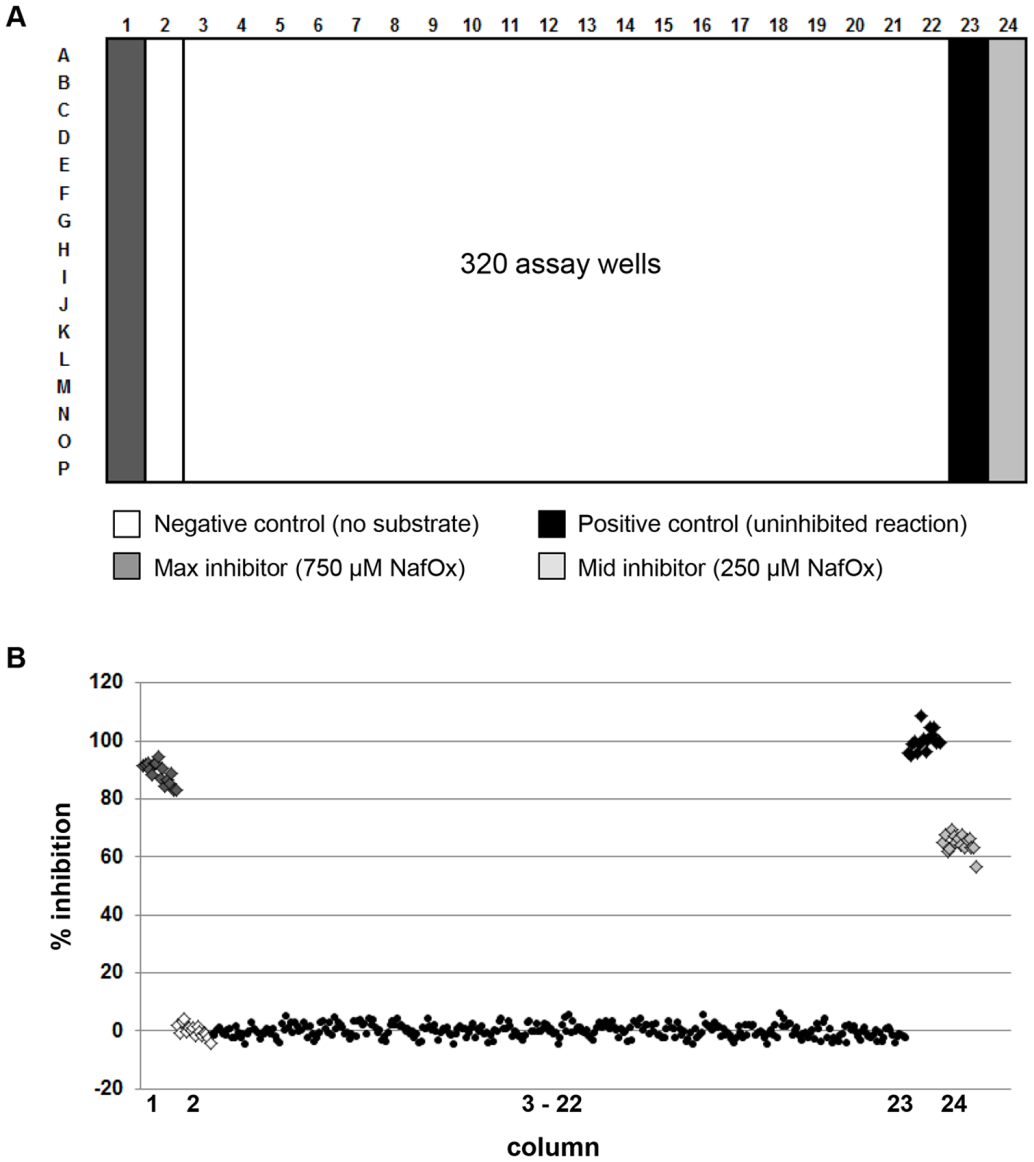
Design and implementation of HTS campaign. **A**) Plate layout for 320-array with four sets of controls (n = 16 for each) in columns 1, 2, 23, and 24. **B**) Results from Blank Plate Validation using DMSO alone in all 320 assay wells.

All validation steps and the final HTS were performed in 384-well plates using a 320 array of compounds (Figure 2A) with the two outside columns on each side of the plate (columns 1, 2, 23 and 24) reserved for appropriate controls, and the central 20 columns for 320 wells of test compounds. As a negative control reaction, to mimic 100% inhibition, we compared reactions conducted without substrate or without enzyme; there was no significant difference between the two. The substrate-free negative control reaction was simpler to incorporate into the HTS flow deck layout/geometry and was chosen as the “maximum inhibition” control. Two different concentrations of control inhibitor (nafronyl oxalate) that consistently displayed ∼50% and ∼80% inhibition of the reaction were placed in the two outer most columns of the plate (1 and 24). The positive controls (full reactions with no inhibition) and negative controls were contained in columns 2 and 23, respectively, in order to minimize any chance of edge-effects. Each of the four controls was performed in 16 wells per plate, a number of replicates that provided powerful statistical evaluation. We confirmed that the reagents were stable in the assay conditions (Text S1). In order to minimize the auto-oxidation of NADH we used a custom-chilling unit maintained at 4°C as a reservoir for NADH-containing solutions. All solutions were freshly prepared every four hours.

Various parameters in the assay were developed and optimized, summarized below and detailed in the Text S1). We created individual programs for each of the separate liquid additions in this assay. This included optimization of the liquid handling steps for each of the robots; adjusting the heights, aspiration and dispensation speeds as well as other protocol details including mixing reagents (Figure S4); and washing of tips. Synchronizing the NADH depletion initiation and the fluorescence generating reaction as tightly as possible reduced variation across a plate. Finally we used the appropriate optic filters in the Victor2 Wallac plate-reader that allowed us to obtain optimal excitation and emission of resorufin with minimal spectral overlap from the light source, and minimal auto fluorescence (details in Text S1).

Validation of HTS compatibility was performed based on NCGC guidelines [14] using the 320-array format described earlier (Figure S1). After confirming low variation of signal across the 320 test wells with uninhibited reactions (Figure 2B, % CV = 2.27) we tested three different concentrations of the control inhibitor, nafronyl oxalate. (2.5 µM, 250 µM and 750 µM) that resulted in a minimum (min), medium (mid) and maximum (max) level of inhibition. Two replicate plates of each, were run on a single day, and repeated on three separate days (for a total of six plates). The resulting % CV across each of the plates ranged from 3.7%–12.9% (Figure S1), acceptable for continuing with the HTS. We performed a final validation of HTS-compatibility by testing a small subset of the LISSP4 library. Three compound plates, each containing 320 compounds, were randomly selected and each was assayed at 5 µM, 10 µM and 20 µM (Figure S2). In addition a 10^th^ plate with no compounds in the 320-array was run alongside these plates. We found that 20 µM showed good levels of inhibition with low background signal. We also saw dose-dependent inhibition from one well, indicating that the pilot screen was successful (Figure S2). The Z′ factor [15] for the controls of each of the three plates tested at 20 µM were 0.803, 0.804 and 0.824 confirming that the assay performance was robust at this concentration.

### Screening LISSP4 Library (27.5 K compounds) and Diversity Library (62.6 K compounds)

Having determined that the fluorescently coupled end-point assay was compatible with high-throughput screening, we proceeded to screen two large compound libraries derived from the Eli Lilly screening collection of >800 K physical samples. The library can be subdivided into a set representing compounds previously identified as actives against human targets (LISSP4 – Lilly Strategic Screening Paradigm 4th iteration) and a set of generally diverse compounds (Diversity 4^th^ iteration) not identified as actives at the time of library construction. LISSP4 contains a representative set of 27582 molecules from active (typically <1 µM) classes of druggable human proteins: proteases, kinases, phosphodiesterases (PDE), GPCRs, nuclear hormone receptors, transporters, etc. Diversity4 contains 62651 molecules representative of compounds from the remainder of the library. The sets were created in November 2008.

**Figure 3:**
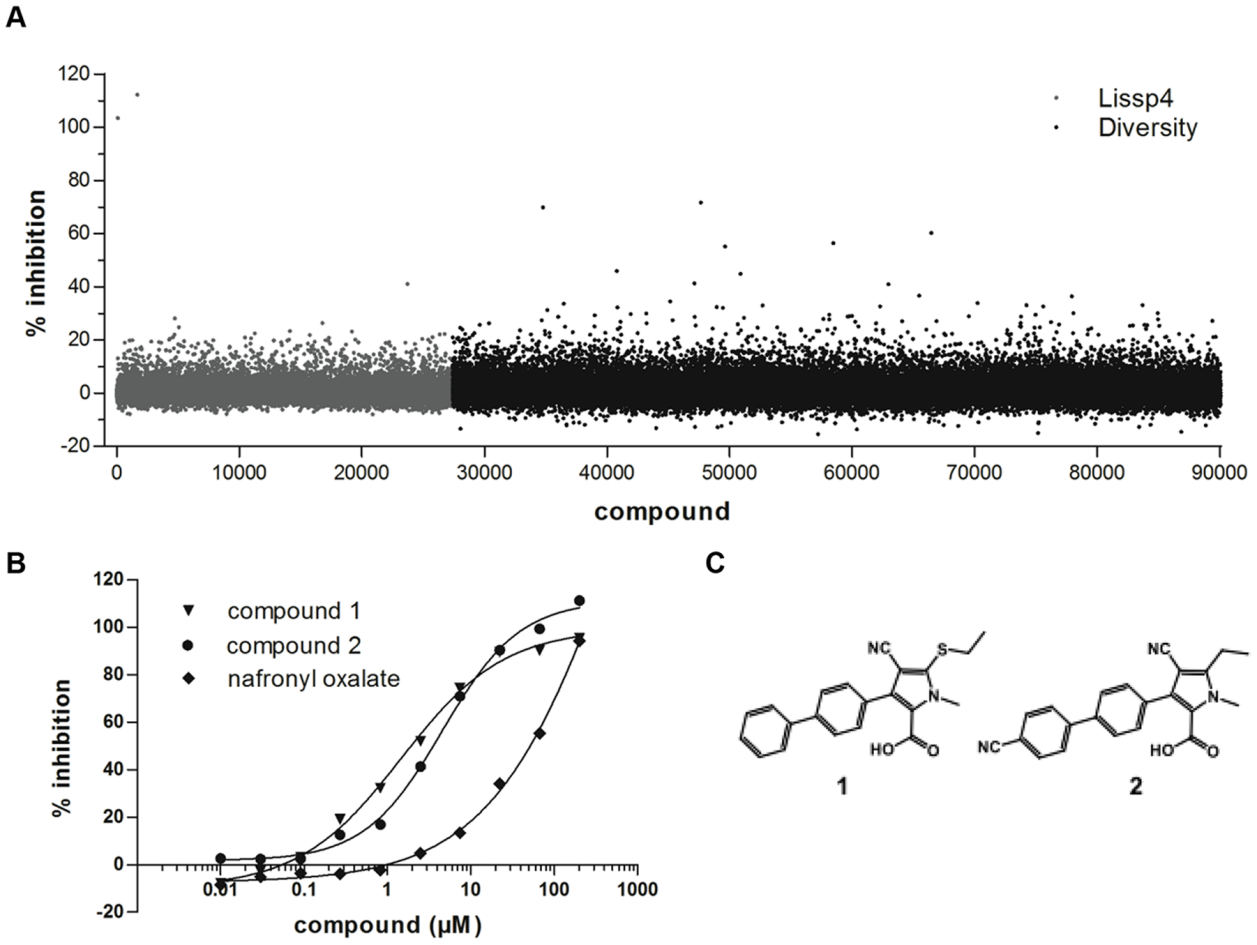
Screening results. **A**) Activity of compounds from LISSP4 (grey) and Diversity (black) libraries shown as percent inhibition against PanC_MTB_. **B**) Concentration response curves (CRCs) for two representative hits and nafronyl oxalate. **C**) Structures of compound **1** and **2**.

The LISSP4 library (27,582 compounds) was screened at a final assay concentration of 20 µM (Figure 3A). Two compounds which showed maximum inhibition were identified as promiscuous inhibitors and were removed from further studies. One compound inhibited 41.1% and eleven compounds inhibited >20%. Many compounds showed modest inhibition (10–20%). Although these had relatively low inhibition in the primary assay a subset of these were considered significant because they inhibited >3 standard deviations away from the mean inhibition across that particular plate.

Although the results from the LISSP4 library confirmed that the assay could identify inhibitors of PanC activity, this library yielded a relatively low hit rate. Therefore, we decided to screen a more diverse compound set, the Diversity library (62,651 compounds) at 20 µM (Figure 3A). Hits were selected as those with inhibition >15% and at least 3 standard deviations higher than the background within that plate. There were more than 180 hit compounds including 6 at >40% and 3 at >60% inhibition. In total there were 222 primary hits from both libraries.

### Confirmation of PanC_MTB_ inhibitory activity

We confirmed our primary hits using two assays. The first was a counter-screen to identify compounds whose activity is due to inhibition of one of the coupling enzymes in the reaction. The second was to run concentration response curves (CRCs).

In addition to the 222 primary hits, the seven most potent primary hits were used as seeds for analog retrieval from the Lilly collection. These seven primary hits comprised 5 distinct classes of compounds; three compounds belonged to the same class. Analog retrieval was performed by chemical fingerprint similarity search and supplemented by substructure searches of identified MedChem Studio scaffold (C2) (Simulation Plus Inc.). This resulted in an additional 78 compounds that were assayed. Of the 300 compounds run against the secondary assays 27 compounds were positive in counter-screen (>10% inhibition), indicating that they targeted one or more of the coupling enzymes in the assay. They were eliminated from further analyses.

The remaining 273 compounds were tested in CRCs against PanC_MTB_. Compounds which were active in CRCs (below 200 µM) were grouped into classes using MedChem Studio scaffold clustering; one class of particular interest was identified. This class contained three active compounds from the primary screen and two analogs retrieved by similarity searches. The two analogs (compounds **1** and **2)** had EC_50_ of 1.8 +/−1.1 µM and 4.0+/−1.1 µM respectively (Figure 3B and C). Compounds of this class did not inhibit the counter screen, and showed dose-dependent inhibition of the PanC_MTB_. The most potent of the four primary hits from this class inhibited PanC in the HTS assay by 71.6% at 20 µM. Tanimoto similarity between the two exemplified hits (**1** vs. **2**) in the fingerprint used in this work space was 0.81.

### Characterization of biochemical enzyme inhibition

**Figure 4:**
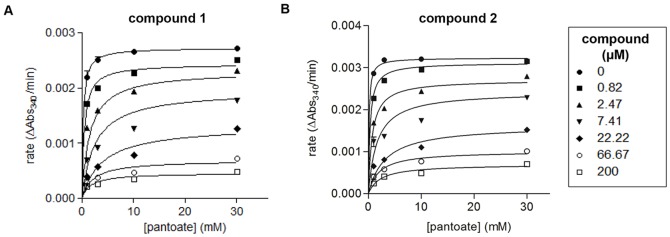
Biochemical characterization of Class 1 compounds. Michaelis-Menten plots with varied concentrations of pantoate and **A**) compound **1** and **B**) compound **2**. Graphpad Prism was used to fit the data to nonlinear regressions.

We used the low-throughput continuous PanC_MTB_ enzyme assay to determine the mode of inhibition, holding all reagents constant but varying a single substrate. Reaction rates were measured as the rate of NADH oxidation and the data were fitted to nonlinear regressions with GraphPad Prism to generate Michaelis-Menten plots (Figures 4A and B). In addition, inhibition constants were determined by nonlinear regression analyses using the general equation of mixed inhibition in GraphPad Prism™, where the resulting parameter “α” determines the mode of inhibition [16]. Both compounds showed non-linear fits closer to competitive inhibition with respect to the substrate pantoate, generating α = 128 and α = 78 for **1** and **2**, respectively. Ki inhibition constants with respect to pantoate were 174+/−20 nM for **1** and 297+/−37 nM for **2**.

### Whole-cell activity against *M. tuberculosis*

**Figure 5:**
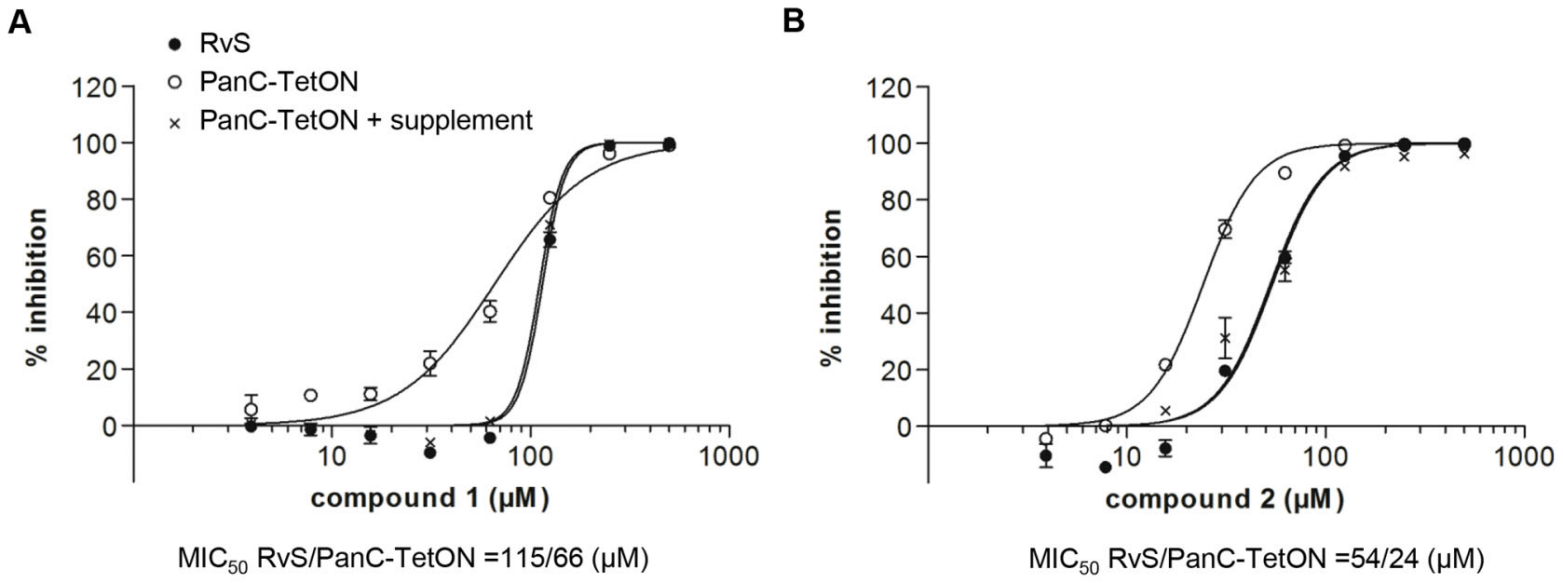
Activity against whole-cell *M. tuberculosis*. Wild-type MTB (RvS; black circles), PanC conditional knockdown (PanC-TetON; white circles), and the knockdown supplemented with pantothenate (PanC-TetON+supplement; x) were grown in the presence of varied concentrations of **A**) compound **1** or **B**) compound **2**. Results are the average and standard deviation of triplicates. The MIC_50_ was determined by plotting %inhibition against compound concentration and fitting to a non-linear curve with Graphpad Prism™.

The compounds were tested for growth inhibitory activity against wild-type *M. tuberculosis* by monitoring ATP levels in cultures exposed to compounds. In each growth condition, bacteria grown in the absence of drug were used as a control reflecting 100% growth. We found that both compounds **1** and **2** were active against live *M. tuberculosis*, with MIC_50_ values of 115 µM and 54 µM respectively (Figure 5). Further, we wanted to ascertain the degree to which this activity could be attributed to the specific inhibition of PanC_MTB_ in whole cells. To do this, we tested the activity of compounds against a conditional PanC-knockdown strain (PanC Tet-ON_M_) in which PanC expression is held under the control of a tetracycline-inducible promoter; in the presence of low concentrations of tetracycline *panC* expression is dramatically reduced [5] and growth is partially attenuated. We used these conditions to maintain growth, while still having artificially lowered PanC levels, making the cell line more sensitive to PanC-mediated inhibition. We found that under lowered *panC* expression the MIC_50_ of both **1** and **2** dropped about two-fold (Figure 5). The MIC_50_ of **1** was reduced from 115 µM in wild-type to 69 µM in the PanC under-expressor strain. Similarly **2** was more potent against the PanC under-expressor strain with an MIC_50_ of 24 µM compared to 54 µM in the wild-type. This suggested that the growth defect is linked to PanC-mediated inhibition. In the presence of higher tetracycline levels *panC* is induced in the PanC Tet-ON_M_ strain but this only partially alleviates the growth defect achieving≈80% of wild-type growth. However, with supplemental pantothenate the pressure caused by the PanC deficiency is relieved and growth is restored to wild-type levels [5]. Therefore to confirm that the hypersensitivity to the PanC inhibitors was not due simply to the attenuated growth, the compounds were also tested against PanC Tet-ON_M_ grown in the presence of pantothenate. As expected, the increased sensitivity was abolished when pantothenate was included in the growth medium, with **1** and **2** displaying MIC_50_ values similar to those in wild type RvS (111 and 53 µM, respectively; Figure 5).

The two compounds were tested for cytotoxicity against the Vero cell line; both showed some toxicity with TC_50_ of 70 µM for compound **1** and 96 µM for compound **2**. We also tested for toxicity against HeLa cells, where the toxicity was less pronounced, with average TC_50_ values of 179 µM and 111 µM respectively.

## Discussion

Several inhibitors of PanC_MTB_ have been identified with varied potency against the enzyme target. A high-throughput screen conducted by Eisenberg and colleagues identified nafronyl oxalate as an inhibitor of PanC_MTB_ with an inhibition constant of ∼75 µM [10], but with no described activity against live *M. tuberculosis*. More potent compounds against PanC_MTB_ have been developed with the tools of structure-based design from fragment screens, and substrate and reaction-intermediate analogs [17]–[19] [20], [21]. The most potent of these displayed nanomolar inhibition of PanC_MTB_, but at the most inhibited bacterial growth by <50% at 128 µM [19]. Most recently a combination of high throughput screening and structure-based design yielded an interesting new inhibitor of PanC_MTB_ [22] whose chemical structure is not related to the enzyme substrates. Actinomycin D (ActD) was first identified as an inhibitor of PanC_MTB_ by screening against the enzyme-based assay [22]. Molecular docking of the ActD cyclopeptide in the active site of PanC_MTB_ was used to identify smaller pharmacophores that might bind in a similar fashion. This revealed one compound with activity against live *M. tuberculosis* (MIC_99_ of ∼54 µM) even though it did not show very high potency in the biochemical assay (IC_50_ = 22 µM).

We sought to identify compounds with novel chemical structures that could serve as scaffolds for drug development targeting PanC_MTB_. We screened a large and diverse compound library, and identified a class of compounds with nanomolar potency against the recombinant purified enzyme. Two compounds were chosen as representative of this class of compounds and were characterized in further detail. Compounds **1** and **2** showed competitive inhibition with respect to pantoate with Ki of 174.1+/−20.0 nM and of 297.1+/−37.1 nM respectively.

In order to ascertain whether the growth inhibition against live *M. tuberculosis* was caused by on-target activity against PanC, we utilized a genetically engineered strain of *M. tuberculosis* in which the expression of PanC is controlled by a tetracycline inducible promoter [5]. This promoter-replacement strategy has been previously used to regulate the expression of target genes in *M. tuberculosis* [23]. Mutants with artificially lowered target expression should be hyper-sensitized to PanC inhibitors, allowing us to infer target-specificity [5], [24]. Moreover, under these conditions a panel of known anti-tubercular drugs had no shifted MIC [5] indicating that the strain is not more susceptible to inhibition of unrelated pathways, and is specifically sensitized to PanC inhibitors. Additionally, the sensitivity gained by lowering the target levels increased our ability to see whole-cell activity of compounds that would otherwise be too weak to be detectable. Both compounds were active against *M. tuberculosis* in an apparently PanC- dependent manner. The MIC_50_ values were high (55 and 118 µM), but a PanC under-expressor was more sensitive and this sensitization was relieved by addition of pantothenate. This suggested the growth inhibitory properties were, at least, partly due to PanC-mediated inhibition.

Both compounds **1** and **2** have a 4-cyano-1-methyl-3-(4-phenylphenyl)pyrrole-2-carboxylic acid core structure. This general compound class has not been studied in depth as antibacterial agents and might provide a unique starting point for tuberculosis drug discovery efforts. These and similar compounds have been reported as a general class of allosteric modulators of the human AMPA receptors [25]. These may still be valuable starting scaffolds whose analogs can be interrogated for improved potency against live *M. tuberculosis* and lowered activity against mammalian cells.

## Supporting Information

Text S1 **Detailed information on tip handling during HTS, compound plating for concentration response curves, reagent stability testing, robotics optimization, and optics for resorufin measurement.**
(DOC)

Figure S1 **Validation of HTS-compatibility assaying plates with max, mid, and min concentrations of a control inhibitor.**
(PDF)

Figure S2 **Data generated in pilot study from one compound plate assayed at three different concentrations.**
(PDF)

Figure S3 **Plate layout for concentration response curves (CRCs).**
(PDF)

Figure S4 **Optimizing well-to-well variation with mixing of solutions by liquid handling equipment.**
(PDF)

## References

[1] WHO (2011) Global tuberculosis control report 2011. Geneva.

[2] Cole ST, Brosch R, Parkhill J, Garnier T, Churcher C, et al (1998) Deciphering the biology of *Mycobacterium tuberculosis* from the complete genome sequence. Nature 393: 537–544. 9634230 10.1038/31159

[3] Sassetti CM, Boyd DH, Rubin EJ (2001) Comprehensive identification of conditionally essential genes in mycobacteria. Proc Natl Acad Sci USA 98: 12712–12717. 11606763 10.1073/pnas.231275498 PMC60119

[4] Bardarov S, Bardarov S Jr, Pavelka MS Jr, Sambandamurthy V, Larsen M, et al (2002) Specialized transduction: an efficient method for generating marked and unmarked targeted gene disruptions in *Mycobacterium tuberculosis*, *M. bovis* BCG and *M. smegmatis* . Microbiology 148: 3007–3017. 12368434 10.1099/00221287-148-10-3007

[5] Abrahams GL, Kumar A, Savvi S, Hung AW, Wen S, et al (2012) Pathway-selective sensitization of *Mycobacterium tuberculosis* for target-based whole-cell screening. Chem Biol 19: 844–854. 22840772 10.1016/j.chembiol.2012.05.020 PMC3421836

[6] Sambandamurthy VK, Wang X, Chen B, Russell RG, Derrick S, et al (2002) A pantothenate auxotroph of *Mycobacterium tuberculosis* is highly attenuated and protects mice against tuberculosis. Nat Med 8: 1171–1174. 12219086 10.1038/nm765

[7] Grassl SM (1992) Human placental brush-border membrane Na(+)-pantothenate cotransport. J Biol Chem 267: 22902–22906. 1429639

[8] Vallari DS, Rock CO (1985) Isolation and characterization of *Escherichia coli* pantothenate permease (*panF*) mutants. J Bacteriol 164: 136–142. 2995306 10.1128/jb.164.1.136-142.1985 PMC214221

[9] Zheng R, Blanchard JS (2001) Steady-state and pre-steady-state kinetic analysis of *Mycobacterium tuberculosis* pantothenate synthetase. Biochemistry 40: 12904–12912. 11669627 10.1021/bi011522+

[10] White EL, Southworth K, Ross L, Cooley S, Gill RB, et al (2007) A novel inhibitor of *Mycobacterium tuberculosis* pantothenate synthetase. J Biomol Screen 12: 100–105. 17175524 10.1177/1087057106296484

[11] Givehchi A, Bender A, Glen RC (2006) Analysis of activity space by fragment fingerprints, 2D descriptors, and multitarget dependent transformation of 2D descriptors. J Chem Inf Model 46: 1078–1083. 16711727 10.1021/ci0500233

[12] Vieth M, Erickson J, Wang J, Webster Y, Mader M, et al (2009) Kinase inhibitor data modeling and *de novo* inhibitor design with fragment approaches. J Med Chem 52: 6456–6466. 19791746 10.1021/jm901147e

[13] Crouch S, Kozlowski R, Slater KJ, Fletcher J (1993) The use of ATP bioluminescence as a measure of cell proliferation and cytotoxicity. J Immunol Meth 160: 81–88. 10.1016/0022-1759(93)90011-u 7680699

[14] NCBI Website (2004) Sittampalam GS, Gal-Edd N, Arkin M, et al., Editors. Assay Guidance Manual [Internet]. Bethesda (MD): Eli Lilly & Company and the National Center for Advancing Translational Sciences; Available: http://www.ncbi.nlm.nih.gov/books/NBK53196/. Accessed: 8 Aug 2013. 22553861

[15] Zhang JH, Chung TDY, Oldenburg KR (1999) A simple statistical parameter for use in evaluation and validation of high throughput screening assays. Journal of Biomolecular Screening 4: 67–73. 10838414 10.1177/108705719900400206

[16] Copeland RA (2002) Tight Binding Inhibitors. Enzymes: John Wiley & Sons, Inc. pp. 305–317.

[17] Hung AW, Silvestre HL, Wen S, Ciulli A, Blundell TL, et al (2009) Application of fragment growing and fragment linking to the discovery of inhibitors of *Mycobacterium tuberculosis* pantothenate synthetase. Angew Chem Int Ed Engl 48: 8452–8456. 19780086 10.1002/anie.200903821

[18] Ciulli A, Scott DE, Ando M, Reyes F, Saldanha SA, et al (2008) Inhibition of *Mycobacterium tuberculosis* pantothenate synthetase by analogues of the reaction intermediate. Chem Biochem 9: 2606–2611. 10.1002/cbic.200800437 PMC4441726 18821554

[19] Velaparthi S, Brunsteiner M, Uddin R, Wan B, Franzblau SG, et al (2008) 5-tert-butyl-N-pyrazol-4-yl-4,5,6,7-tetrahydrobenzo [d]isoxazole-3-carboxamide derivatives as novel potent inhibitors of *Mycobacterium tuberculosis* pantothenate synthetase: initiating a quest for new antitubercular drugs. J Med Chem 51: 1999–2002. 18335974 10.1021/jm701372r

[20] Wang S, Eisenberg D (2003) Crystal structures of a pantothenate synthetase from *M. tuberculosis* and its complexes with substrates and a reaction intermediate. Prot Sci 12: 1097–1108. 10.1110/ps.0241803 PMC2323879 12717031

[21] Wang J, Eisenberg D (2006) Crystal structure of the pantothenate synthetase from *Mycobacterium tuberculosis*, snapshots of the enzyme in action. Biochemistry 45: 1554–1561. 16460002 10.1021/bi051873e

[22] Yang Y, Gao P, Liu Y, Ji X, Gan M, et al (2011) A discovery of novel *Mycobacterium tuberculosis* pantothenate synthetase inhibitors based on the molecular mechanism of actinomycin D inhibition. Bioorg Med Chem Lett 21: 3943–3946. 21641210 10.1016/j.bmcl.2011.05.021

[23] Ehrt S, Guo XV, Hickey CM, Ryou M, Monteleone M, et al (2005) Controlling gene expression in mycobacteria with anhydrotetracycline and Tet repressor. Nucl Ac Res 33: e21. 10.1093/nar/gni013 PMC548372 15687379

[24] Wang J, Soisson SM, Young K, Shoop W, Kodali S, et al (2006) Platensimycin is a selective FabF inhibitor with potent antibiotic properties. Nature 441: 358–361. 16710421 10.1038/nature04784

[25] Fernandez MC, Castaño A, Dominguez E, Escribano A, Jiang D, et al (2006) A novel class of AMPA receptor allosteric modulators. Part 1: design, synthesis, and SAR of 3-aryl-4-cyano-5-substituted-heteroaryl-2-carboxylic acid derivatives. Bioorg Med Chem Lett 16: 5057–5061. 16879964 10.1016/j.bmcl.2006.07.035

