# Supplementary material for: A High-Throughput Screen against Pantothenate Synthetase (PanC) Identifies 3-Biphenyl-4-Cyanopyrrole-2-Carboxylic Acids as a New Class of Inhibitor with Activity against Mycobacterium tuberculosis
Source: PLoS One. 2013 Nov 7;8(11):e72786. doi: 10.1371/journal.pone.0072786 (PMC3820577; doi:10.1371/journal.pone.0072786)
Supplement: Text S1 — Detailed information on tip handling during HTS, compound plating for concentration response curves, reagent stability testing, robotics optimization, and optics for resorufin measurement. (DOC) [file pone.0072786.s001.doc]

**SUPPORTING INFORMATION TEXT S1**

**Tip handling during HTS:**

In order to minimize the number of tips used during screening the 96 tip array was washed using a custom tip washer operated by a software script. The tips were washed externally and internally in 96 individual fountains of fluid with a series of aspiration and dispense steps. The tips were then dried with a vacuum. The 384 tip array was also washed through a series of aspiration and dispense steps, but instead of a fountain system, washes were performed with aspiration and dispense steps in two reservoirs containing wash fluid. Any trace fluid retained on the tips after the completion of the wash cycles was dispensed out of the tips to blotter paper by a pre-wash air gap.

**Compound plating for concentration response curves:**

We developed a precision compound dilution method for use in the PanC assay so that the CRCs for an individual compound were conducted within a plate rather than between multiple plates. Compounds in 100% DMSO were plated at 10 mM in columns 3 and 13. We developed liquid handling programs to perform precise 3-fold serial dilutions of compounds across 10 wells of the plate in 100% DMSO. The DMSO stock plate was then used to prepare the aqueous compound dilution plate. This template resulted in each compound being tested from 200 µM to 10 nM within a single assay plate (Supporting Figure S3). Two published inhibitors of PanC were included in each plate as additional controls.

**Reagent stability testing:**

The stability of the reagent mixtures was tested over the course of 6 hours at RT and at 4^o^C. The rate of the reaction was not affected by leaving the reagents at RT for up to 4 hours, indicating that the enzymes in the solution are stable. However, we did notice that while the enzymes seem stable, the βNADH substrate in the reaction degraded rapidly at RT. Although this did not affect the initial rate of the kinetic reaction, it altered the total amount of βNADH available for the reaction, and hence the fluorescence readout that was directly dependent on the total βNADH in solution. In contrast, the βNADH-containing solution was stable at 4^o^C for up to 6 hours. Therefore we used a custom chilling unit maintained at 4^o^C as a reservoir for the βNADH-containing enzyme solution.

**Robotics optimization:**

Two liquid handling robots were used: a NanoScreen 384-well head and a 96-well head MultiMek liquid dispensing robot. The assay was performed by first plating 20 µL of the βNADH-containing enzyme solution into all wells of the plate using the 96 well MultiMek liquid dispensing robot. The tips were washed and dried and used to transfer 5 µL of compound from one quadrant of the 384-well compound dilution plate into the corresponding quadrant of the 384-well assay plate. The tips were washed and dried by aspiration between each of the four quadrants. The plate was then moved to the NanoScreen 384-well head liquid dispensing robot where the enzyme cascade initiating βNADH-depletion was activated by a single addition of the PanC substrates (pantoate and β-alanine). The plate was returned to the plate-holding carousel for 30 minutes. Following incubation the plate was brought to the NanoScreen 384-well head liquid handling robot, the fluorescence reagents were dispensed across the entire plate in a final liquid addition, and the plate was returned to the carousel. After 5 minutes the plate was manually taken to a plate-reader and the fluorescence generated by the βNADH dependent reaction was measured.

Careful scheduling routines were designed with the SAMI software so that each plate moving through the automation received the same temporal conditions. One variable that greatly enhanced the synchronization of the reactions across the plate was the use of a 384-head liquid dispensing robot during two liquid addition steps; the addition of the enzyme-cascade initiating substrates and the final addition of fluorescence reagents. A second variable that aided in synchronizing the reactions across the plate was the addition of a mixing step. We determined that the coefficient of variance (CV) between wells was dramatically improved with 3 mixes (CV = 8.93% and 29.7% for 3 mixes and no mixes, respectively, n=96 wells; Supporting Figure S4). However, excessive mixing resulted in occasional liquid residue in some of the tips and a corresponding increase in the CV (CV = 19.63% for 5 mixes, n=96 wells). Therefore we included two automated mixing steps in the assay: one after addition of the substrate solution, to initiate the βNADH oxidation enzyme cascade, and the second after addition of the fluorescence reagents, both performed with the 384-head liquid dispensing robot.

In addition to optimizing the robotics for this assay, we included a short assay of the reagents to optimize the incubation period prior to addition of the fluorescence reagents. This protocol was used to test the PanC assay in a few wells prior to commencing the full screen. Because each solution preparation varied slightly in their final concentrations, we found it useful to confirm that all the reagents were prepared correctly and that the assay ran as anticipated. In addition, this pre-HTS assay optimized the reproducibility of the final assay by confirming a precise incubation time for the PanC assay (typically set to 30 minutes) prior to fluorescence coupling. The pre-HTS assay was performed using the same liquid handling programs as the final HTS, except that the final addition of the fluorescent reagents was performed manually, to allow for collection of data in 5 minute intervals.

**Optics for resorufin measurement:**

Prior publications have shown that the peak excitation and emission wavelengths for resorufin can vary[^16^](#_ENREF_16). Therefore to determine the optimal filters to be used in our assay we measured the excitation and emission spectra of resorufin using Biotek Synergy 4 monochromator settings by first fixing the excitation wavelength close to the published peak value (543 nm) and scanning the emission spectra (570 nm - 620 nm). We next fixed the emission wavelength (605 nm) and scanned the excitation spectra (460 nm -578 nm). From this study we determined that the peak excitation and emission of resorufin was at 570 nm and 590 nm, respectively. The appropriate optic filters that were available for the Victor2 Wallac plate-reader had an excitation filter set at 560 nm with an 8 nm bandwidth and an emission filter set at 590 nm with a 10 nm bandwidth. This allowed us to obtain optimal excitation and emission with minimal spectral overlap from the light source. This spectral range also minimizes auto fluorescence.
