# Supplementary figures and images for: A High-Throughput Screen against Pantothenate Synthetase (PanC) Identifies 3-Biphenyl-4-Cyanopyrrole-2-Carboxylic Acids as a New Class of Inhibitor with Activity against Mycobacterium tuberculosis
Source: PLoS One. 2013 Nov 7;8(11):e72786. doi: 10.1371/journal.pone.0072786 (PMC3820577; doi:10.1371/journal.pone.0072786)

# Supporting Figure S1

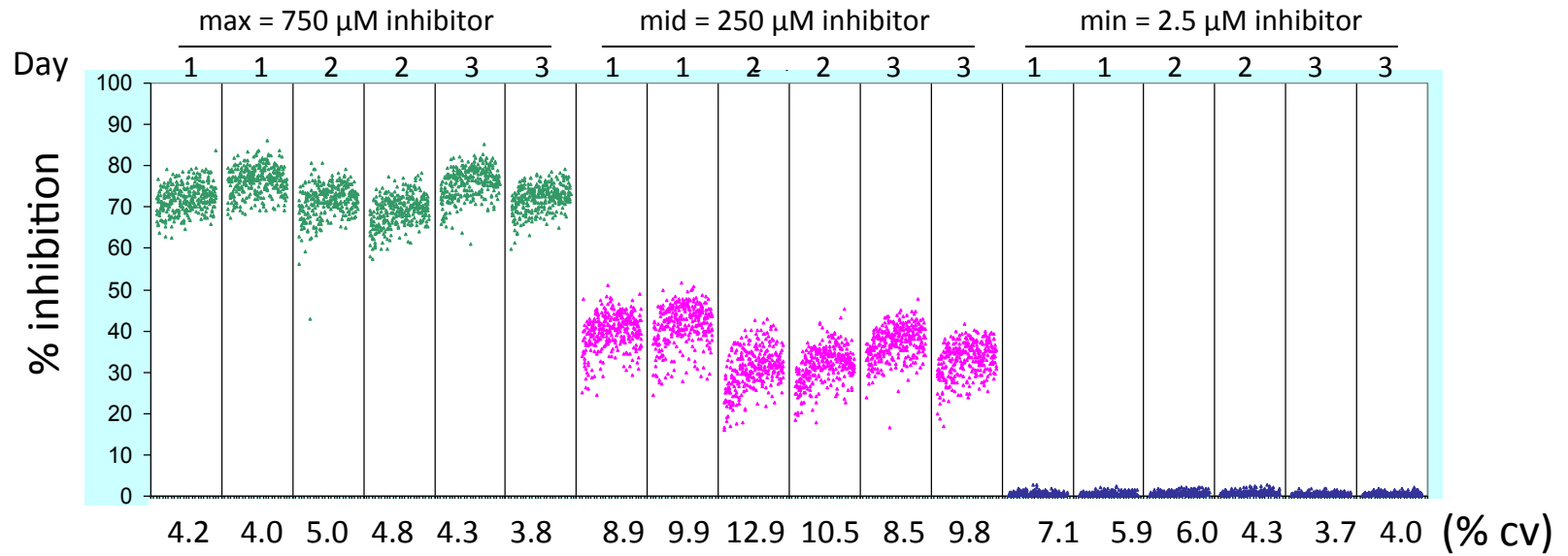

Supplement: Figure S1 — Validation of HTS-compatibility assaying plates with max, mid, and min concentrations of a control inhibitor. (PDF) [file pone.0072786.s002.pdf]

## Supporting Figure S2

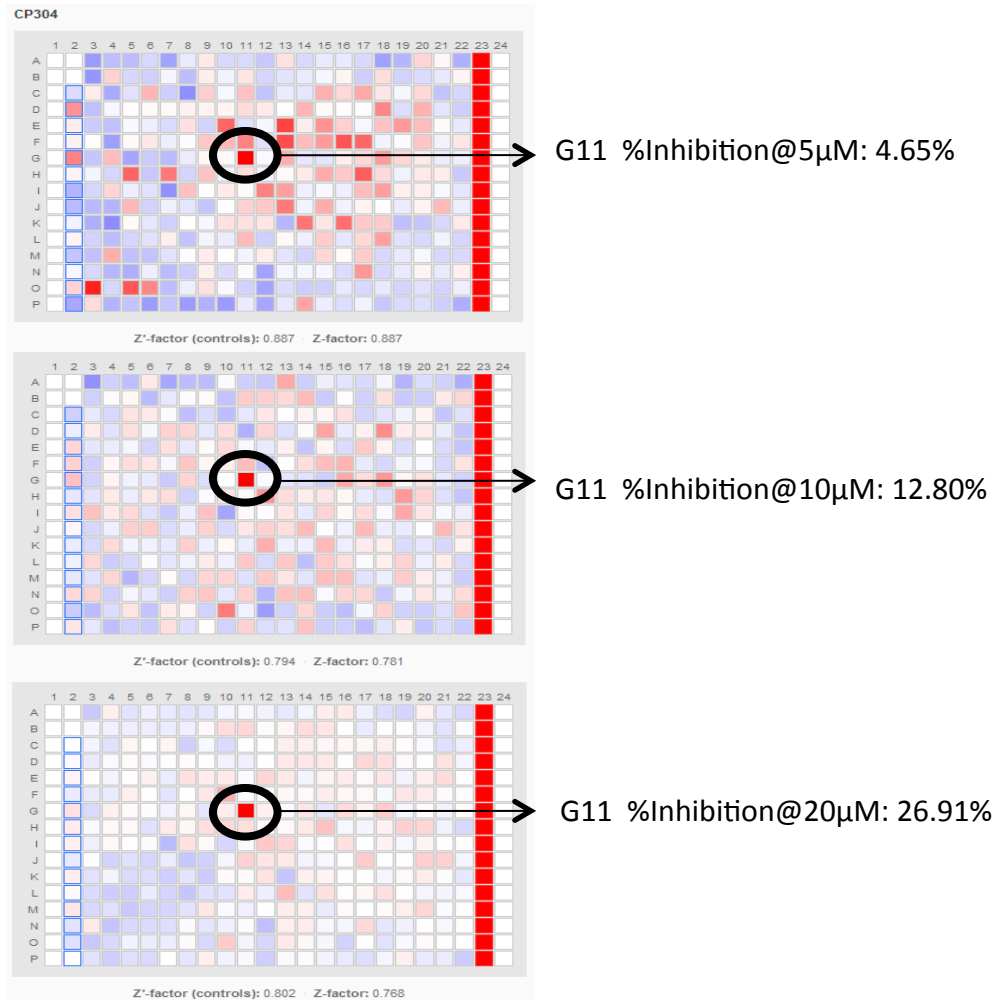

Supplement: Figure S2 — Data generated in pilot study from one compound plate assayed at three different concentrations. (PDF) [file pone.0072786.s003.pdf]

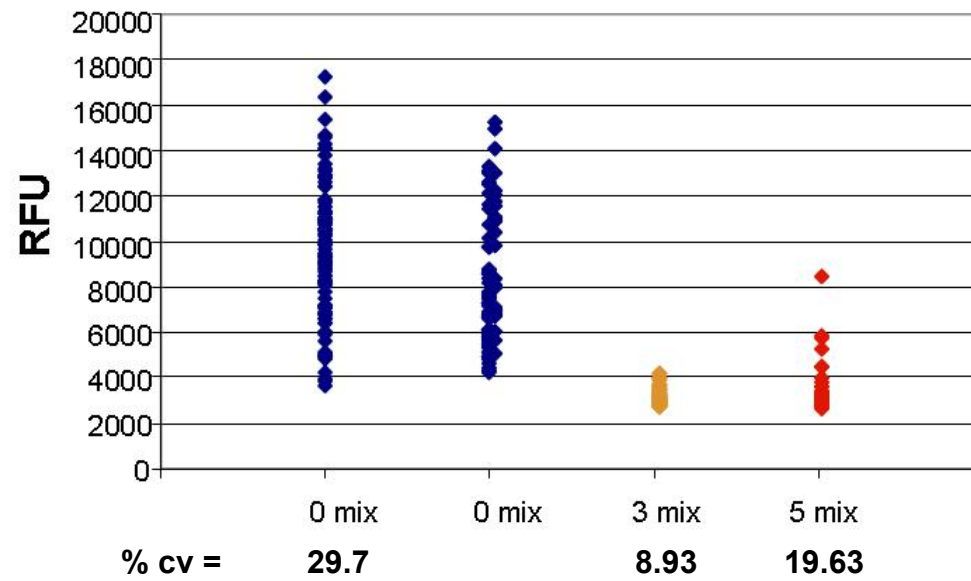

Supplement: Figure S4 — Optimizing well-to-well variation with mixing of solutions by liquid handling equipment. (PDF) [file pone.0072786.s005.pdf]
