# Supplementary material for: A High-Throughput Screen against Pantothenate Synthetase (PanC) Identifies 3-Biphenyl-4-Cyanopyrrole-2-Carboxylic Acids as a New Class of Inhibitor with Activity against Mycobacterium tuberculosis
Source: PLoS One. 2013 Nov 7;8(11):e72786. doi: 10.1371/journal.pone.0072786 (PMC3820577; doi:10.1371/journal.pone.0072786)

Supporting Figure S3

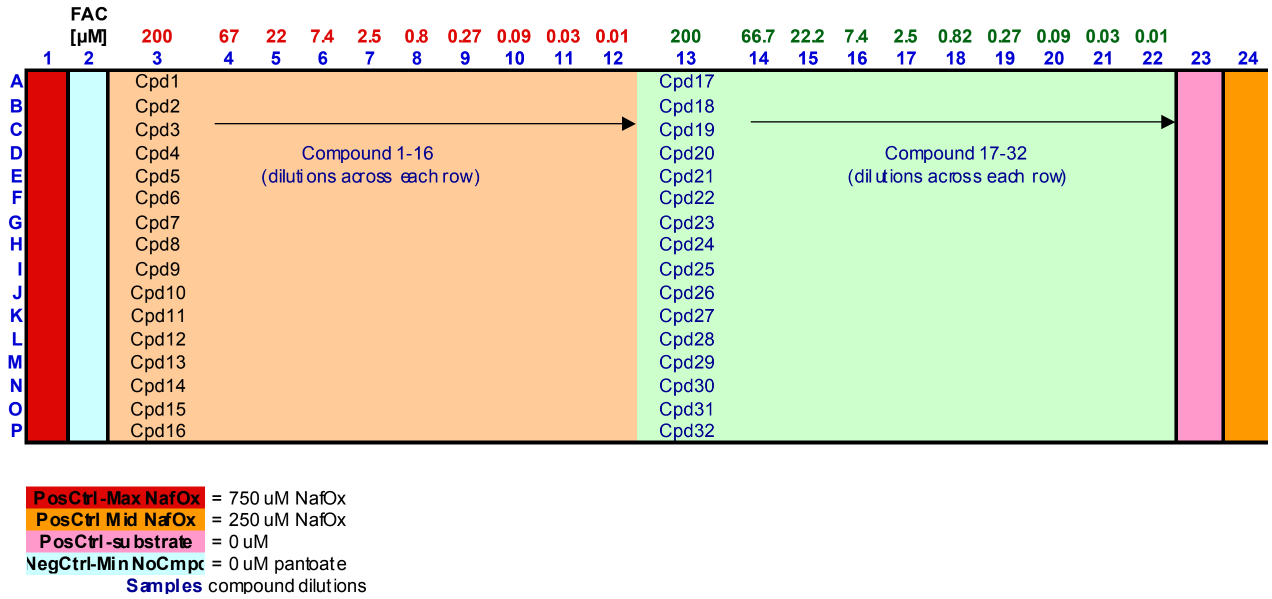

Supplement: Figure S3 — Plate layout for concentration response curves (CRCs). (PDF) [file pone.0072786.s004.pdf]
